# Early Developmental Trajectories in Infants With Neurofibromatosis 1

**DOI:** 10.3389/fpsyg.2022.795951

**Published:** 2022-07-22

**Authors:** Shruti Garg, Ming Wai Wan, Jannath Begum-Ali, Anna Kolesnik-Taylor, Jonathan Green, Mark H. Johnson, Emily Jones

**Affiliations:** ^1^Division of Neuroscience and Experimental Psychology, Manchester Academic Health Science Centre, University of Manchester, Manchester, United Kingdom; ^2^Royal Manchester Children’s Hospital, Central Manchester University Hospitals NHS Foundation, Manchester, United Kingdom; ^3^Division of Psychology and Mental Health, School of Health Sciences, Manchester Academic Health Science Centre, University of Manchester, Manchester, United Kingdom; ^4^Centre for Brain and Cognitive Development and Department of Psychology, Birkbeck, University of London, London, United Kingdom; ^5^MRC Cognition and Brain Sciences Unit, University of Cambridge, Cambridge, United Kingdom; ^6^Department of Psychology, University of Cambridge, Cambridge, United Kingdom

**Keywords:** NF1 = neurofibromatosis type 1, autism, natural history, cognition, behaviour

## Abstract

**Objective:**

To examine the trajectories of cognitive, motor and behavioural development in infants with NF1 compared to infants without a family history of neurodevelopmental difficulties.

**Study design:**

Infants with NF1 and low-risk controls were recruited from 5 months of age and followed longitudinally. Data from standardised tests was gathered at 5, 10 and 14 months and developmental trajectories of motor, language, behaviour, sleep, social development and parent–infant interaction were examined. Linear mixed modelling was used to estimate group differences in cognitive and behavioural measures over time.

**Results:**

No group differences were observed on Mullen Scale of Early Learning, overall adaptive functioning, temperament or behavioural measures. There were no group differences observed on measures of social communication or parent–infant interaction. Over the course of development, the NF1 group slept less and took more time to settle to sleep as compared to the control group. Maternal education was significantly associated with cognitive and behavioural developmental outcomes in both groups.

**Conclusion:**

Cognitive, social and behavioural impairments are a cause of significant functional morbidity in children with NF1. This report is the first study to investigate the trajectories of cognitive, motor and behavioural development in infancy in NF1. Our results demonstrate that overall cognitive and behavioural developmental trajectories of the NF1 group in the infancy period are similar to controls. Given previous reports of delayed development in the NF1 cohort by 40 months, early clinical interventions strategies to promote sleep hygiene may be beneficial to optimise developmental outcomes.

## Introduction

Neurofibromatosis 1 is a common autosomal dominant single gene neurodevelopmental disorder, with birth incidence of 1:2700 (Evans et al., 2010). Approximately 50% of the cases are inherited and the rest are caused by a *de novo* mutation of the NF1 gene on chromosome 17q11.2, which has an important role in intracellular signalling, learning and synaptic plasticity (Costa et al., 2002). Diagnosis of NF1 may be established if two out the seven National Institute of Health (NIH) defined clinical criteria are met; these include (i) presence of ≥6 café-au-lait macules, (ii) ≥2 neurofibromas or ≥, (iii) freckling in the inguinal or axillary regions, (iv) optic pathway glioma, (v) ≥2 Lisch nodules, (vi) a distinctive osseous lesion, and (vii) a first- degree relative with NF1. In families with history of NF1, genetic tests on umbilical cord blood may be used to diagnose NF1 early in infancy. Whilst the disorder is known for its cutaneous manifestations, substantial morbidity in the paediatric population is due to cognitive, social and behavioural impairments. Specific learning impairments are common, although overall cognitive ability or IQ is in the low average range, with approximately 5–10% of individuals in the learning disability range (Lehtonen et al., 2015). Studies suggest a high prevalence of both ASD of approximately 25% and ADHD of about 50% (Garg et al., 2013).

Our understanding of the emergence of behavioural phenotype in NF1 is limited. Much of what is known is drawn from cross-sectional studies in school-age children with only handful of studies in the preschool population (Brei et al., 2014; Klein-Tasman et al., 2014; Lorenzo et al., 2015). These studies suggest that the NF1 gene mutation confers a general vulnerability for cognitive, motor and language difficulties that is observable in the preschool period. In a study of 40 children with NF1, aged 3–6 years compared to matched controls, impairment in at least one area (verbal, non-verbal or spatial skills) was found in 45% of the sample (Klein-Tasman et al., 2014). Similarly, a cross-sectional study of 39 toddlers aged 21–30 months with NF1 found poorer cognitive, motor and language development in the NF1 group compared to age matched controls. Mean cognitive development in the NF1 group was one standard deviation below controls, with a third of the NF1 cohort with low average motor development and parental responses indicated delayed receptive and expressive language development in over 70% (Lorenzo et al., 2011). Brei et al. (2014) assessed language abilities in 30 children with NF1 aged 4–6 years and found impairments in core language skills in a third of the sample, which were not fully accounted for by attentional impairments (Brei et al., 2014).

Whilst these cross-sectional studies suggest a vulnerability to cognitive impairment in the preschool period, little is known about the developmental trajectories of these behavioural phenotypes. Understanding the natural history of development in NF1 is important for several reasons. Longitudinal studies can offer insights into brain development and identify early predictors of later neurodevelopmental outcome. Further, neurodevelopmental disorders comorbid with NF1 such as ASD and ADHD, which are diagnosed based on behavioural assessments in school-age children likely emerge through a complex developmental cascade of interactions between the gene, brain, behaviour and the child’s interaction with the environment. Early interventions targeted in the prodromal period (before behavioural symptoms of ASD and ADHD emerge) could ameliorate the later emergence of behavioural symptoms (Green et al., 2017). Targeted pharmacological treatments reverse the NF1 associated cognitive impairments in animal models but translational clinical trials in humans have so far had mixed results (van der Vaart et al., 2013; Stivaros et al., 2018). Understanding the developmental trajectories in NF1 will allow identification of treatment-sensitive early markers as surrogate endpoints in treatment trials. Lastly, diagnosis and intervention approaches in NF1 are complicated by the inter- and intra-familial variability in phenotypic expression (Sabbagh et al., 2009). Mapping individual developmental trajectories of development may provide insight into causal mechanisms and the role of modifying genes in phenotypic expression in NF1.

Two preliminary studies have examined trajectories of symptoms in NF1 in toddlerhood. In a longitudinal study of 39 children with NF1 and matched control assessed at 21, 30 and 40 months, the trajectory of cognitive development in NF1 diverged from the control groups over time, and early productive vocabulary was a significant predictor of later language skills (Lorenzo et al., 2015). Using parental reports of developmental progression, Wessel et al. (2013) reported that children aged 0–8 years with NF1 shifted between delays and typical performance in all areas of cognitive functioning, and showed persistent gross motor function delays from toddlerhood (Wessel et al., 2013). Both studies illustrate cognitive differences in NF1 emerge as early as the second year of life, but little is known about how these trajectories evolve in infancy. The aim of the present study was to examine the natural history of cognitive, motor and behavioural development in infants with NF1 compared with a group of low-risk infants with no familial risk of neurodevelopmental difficulties. Our primary objective was to determine how NF1 genetic variance might manifest in early development including in cognitive, social and behavioural development. A preliminary case series (Kolesnik et al., 2017) published from a subset of the current sample of infants at 10 months indicated early emerging differences in language and motor skills, confirming the importance of studying developmental abilities in the first year of life. Here we examine longitudinal trajectories of development from 5 to 14 months in a larger sample of infants, compared to low-risk controls. Based on previous studies, we expected that the infants with NF1 would have lower motor and early language development as compared to the control group.

## Materials and Methods

The Early DEvelopment in NF1 (EDEN) study is a prospective longitudinal study of infants with NF1. Participants were recruited via regional genetic centres and national NF charities in the United Kingdom. Inclusion criteria for the NF1 group included (i) infant <12 months at time of recruitment, (ii) confirmed diagnosis of NF1 via molecular testing of cord blood samples or clinical diagnosis based on NIH consensus criteria (National Institutes of Health Consensus Development Conference, 1988). Inclusion criteria for the control group were (i) infants <12 months with no first-degree relatives with a diagnosis of ASD or ADHD or known genetic disorders; (ii) no developmental concerns reported by parents; (iii) full-term birth (gestational age greater than 36 weeks). Participants in the control group were recruited from a volunteer database at the Centre for Brain and Cognitive Development, Birkbeck, University of London (STAARS study). Exclusion criteria for both groups included (i) conditions that may make harder for an infant to participate (including any serious physical complications due to NF1 as judged by the referring clinician), (ii) significant vision or hearing abnormalities, (iii) significant prematurity, (iv) parents with evidence of significant learning difficulties or who are unable to give informed consent. The NF1 and the control groups over a 4-year period between 2016 and 2019. The sample size calculation was based on our previous studies comparing infants at high familial risk for ASD to controls [e.g., *n* = 17 (Elsabbagh et al., 2013), η^2^ = 0.17; *n* = 19 (Elsabbagh et al., 2009), η^2^ = 0.16] and was based on detecting differences on EEG biomarkers rather than behavioural measures as reported in this study.

### Procedure

The study assessment took place at the Centre for Brain and Cognitive Development, Birkbeck, University of London. Written informed consent was provided by the parent prior to the commencement of the study. The testing only took place if the infant was in a content and alert state. Participant families were reimbursed expenses for travel, subsistence and overnight stay if required. Behavioural measures described below were administered as part of a more extensive experimental protocol.

### Measures

Cognitive ability at 5, 10 and 14 months was assessed through the *Mullen Scales of Early Learning* (MSEL), an observational measure that assesses gross and fine motor skills, expressive and receptive language, and visual reception; analyses used raw scores for each domain (Mullen, 1995). Adaptive skills were assessed at 5, 10 and 14 months using the *Vineland Adaptive Behaviour Scale* (VABS), a parent-report measure that assesses socialisation, communication, motor behaviour and daily living skills and provides an overall composite score referred to as Adaptive Behaviour Composite (ABC); analyses used standard scores per domain (Sparrow, 2011). Language development at 10 and 14 months was assessed using parent reported MCDI (Fenson et al., 2007). The questionnaire is comprised of approximately 800 items, summed to produce a receptive and expressive vocabulary score. The resulting score was log transformed to base 10 for analyses. Autism symptoms were assessed using the AOSI, a 19-item interactive observation schedule designed to capture early signs of ASD and measures aspects of visual attention, social-communication, development of sensory and motor skills. Absence/presence of behaviours is rated 0–3, where 0 signifies typical function, and higher values suggests increasing deviation from the typical behaviour expected at the age of assessment (Bryson et al., 2008). Temperament was assessed at 5, 10 and 14 months using IBQ a widely used parent-report measure comprised of 14 subscales, grouped into three factors: Surgency (child’s tendency to show excitement, positive affect and approach), Negative Affect (tendency to cry, be avoidant or otherwise fussy), and Regulation Capacity (ability to regulate their mood and behaviour) (Putnam et al., 2014). The SSQ (Matthey, 2001) was used to assess parental perception of infant sleep patterns. Parental reports of total sleep duration, number of night awakenings and time taken to settle (in minutes) are reported in this study. Parent–infant interaction was assessed at the 10- and 14-month visits using the MACI (Wan et al., 2017). A video-recorded caregiver–infant free play interaction session with toys was coded by two independent raters, blinded to family information. The coding scheme comprised of eight 7-point scales, from which we focused on four scales of areas of parent–infant interaction that were affected among infants with familial likelihood of ASD (Wan et al., 2013): caregiver sensitivity, caregiver non-directiveness, infant attentiveness to caregiver, and dyad mutuality (the amount and degree of dyadic reciprocity, closeness and sharedness). Maternal education was classified as primary, secondary, undergraduate or postgraduate. Further details of this measure and inter-rater reliability are provided in the Supplementary Materials.

### Statistical Analyses

Statistical analyses were performed using R version 3.6 (R Core Team, 2019). Linear mixed modelling was used to estimate change in the cognitive and behavioural measures across time using the ‘nlme’ package. For each outcome, the overall group differences were modelled using fixed effects of predictors (group, participant age in days at time of assessment and maternal education) and random effect of individual variation. Because of the sample size, predictors and interactions were limited and models were of the general form: *Model*<*- lme (outcome*∼ age*group + *maternal education, random* = *1| ID*). Maternal education was included as a predictor as it is strongly associated with cognitive development (Jackson et al., 2017). The control group were treated as baseline and parameters were estimated for the NF1 group. Missing data were handled with the maximum likelihood approach.

A *p*-value of <0.05 was considered significant. Removal of maternal education from the models did not alter overall results.

## Results

**FIGURE 1:**
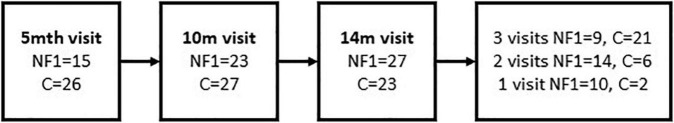
Flowchart showing the number of NF1 and Control (C) infant participants at each time-point in the study.

Thirty-three infants with NF1 and 29 typically developing infants were enrolled. Due to flexible enrolment and variability in compliance, the sample size at each time point varied as shown in Figure 1.

At the 5-month assessment, the NF1 group was significantly older than the control group (*t* = −3.09, *p* = 0.004) but there were no significant age differences between the groups at 10- or 14-month assessments. There was a significant difference in maternal education with mothers in the control group more likely to have a graduate or post graduate education (Median NF1: 2, Control: 4 χ^2^ = 24.78, *p* < 0.001). There was no significant sex difference between the groups at any time point. Within the NF1 group, NF1 was inherited in 30 participants; *de novo* in 2 participants and inheritance was unknown in 1 participant. Further details of the demographic, clinical characteristics are provided in Table 1.

**TABLE 1:** Descriptive statistics including means and SDs for the NF1 and control group at the three assessment time points.

	5 months				10 months				14 months			
	Control group		NF1		Control group		NF1		Control group		NF1	
	*n*	*Mean (SD)*	*n*	*Mean(SD)*	*n*	*Mean(SD)*	*n*	*Mean(SD)*	*n*	*Mean(SD)*	*n*	*Mean(SD)*
Age (days)	26	179.19(14.05)	15	194.73(17.85)	27	321.93(16.70)	23	327.0(17.11)	23	447.61(18.42)	27	449.74(23.41)
Male gender	26	18	15	8	27	16	23	12	23	13	27	13
Maternal education (*n*) (secondary/undergrad/postgrad)	24	2/9/13	11	7/2/2	26	2/10/14	22	14/5/3	23	2/7/14	25	14/8/3
**Mullen Scale of Early Learning T scores**												
*Gross motor*	26	43.69(9.41)	15	37.40(9.73)	27	34.89(11.78)	23	30.0(10.25)	23	36.74(13.37)	27	31.96(12.75)
*Visual reception*	26	47.27(6.33)	15	37.47(11.53)	27	48.85(7.99)	23	43.96(8.08)	23	35.09(8.88)	27	33.67(5.17)
*Fine motor*	26	42.92(10.46)	15	32.80(6.99)	27	51.63(12.88)	23	42.13(11.29)	23	49.65(12.18)	27	43.85(11.07)
*Receptive language*	26	36.88(11.28)	14	25.71(9.31)	27	39.26(8.96)	23	34.87(7.73)	23	32.87(6.50)	27	28.22(6.01)
*Expressive language*	26	41.85(7.45)	15	35.13(6.35)	27	36.85(9.89)	23	39.95(12.57)	23	37.09(8.80)	27	38.19(10.18)
**Vineland Adaptive Behaviour Scale**												
*Communication*	22	94.95(10.91)	14	95.71(18.26)	21	92.86(12.76)	23	86.13(14.91)	19	96.11(10.49)	24	91.04(10.08)
*Daily living skills*	22	86.32(19.86)	14	92.71(18.26)	22	102.45(10.76)	23	93.74(9.14)	18	93.44(8.39)	23	95.52(10.85)
*Socialisation*	23	98.70(12.26)	14	101.36(14.21)	22	98.77(10.04)	21	92.76(14.59)	18	94.22(9.79)	23	97.96(9.43)
*Motor*	21	88.67(12.99)	14	85.07(13.37)	21	95.76(16.86)	23	76.91(14.86)	18	101.67(9.49)	24	93.42(11.13)
*Adaptive behaviour composite*	19	90.63(11.92)	14	92.29(11.13)	20	96.80(10.44)	21	85.86(12.85)	17	95.29(9.73)	23	95.29(9.36)
**MacArthur Communication Inventory**												
*Receptive vocabulary*					21	15.38(15.52)	22	36.05(48.97)	19*^a^*	77.47(68.37)	22*^a^*	74.64(72.61)
*Expressive vocabulary*					21	0.33(0.97)	22	1.18(1.99)	19*^b^*	10.53(13.09)	23	7.96(10.52)
**Infant Behaviour Questionnaire**												
*Surgency*	17	4.16(0.82)	10	4.42(0.77)	22	4.80(0.46)	21	4.83(0.72)	19	5.18(0.60)	25	4.96(0.59)
*Negative affectivity*	21	3.17(0.72)	14	2.96(0.68)	21	3.50(0.66)	21	3.73(1.08)	18	3.43(0.66)	26	3.7(1.00)
*Regulation capacity*	19	5.09(0.51)	13	5.08(0.67)	20	4.76(0.47)	22	4.80(0.89)	19	4.80(0.67)	26	4.79(0.76)
**Sleep and Settle Questionnaire**												
*Sleep at night (Min)*	25	454.80(194.55)	11	480.0(228.57)	22	555.5(185.86)	22	479.32(212.86)	19	570.26(136.14)	22	469.77(149.24)
*Number of night wakes*	25	2.5 (1.45)	12	2.62(1.85)	22	1.32 (1.20)	23	1.72(2.09)	19	1.11(1.50)	24	1.60(1.34)
*Time taken to settle*	24	15.81(24.79)	12	23.20(49.63)	22	11.79(13.36)	20	17.47(16.76)	19	8.40(10.60)	23	16.74(16.88)
*Total confidence*	25	9.36(1.36)	13	9.23(1.36)	22	9.09(1.27)	23	9.00(1.35)	19	9.53(0.70)	25	9.04(1.17)
AOSI total score									18	10 (5.13)	24	12.13(4.88)
**MACI**												
*Sensitive responsiveness*					22	4.50(0.96)	19	3.68(1.16)	16	4.75(1.18)	19	4.21(1.08)
*Non-directiveness*					22	4.59(0.96)	19	4.0(1.11)	16	4.88(1.20)	19	4.21(1.08)
*Infant attentiveness*					22	4.55(1.22)	19	3.58(1.17)	16	4.94(1.06)	19	4.37(1.21)
*Mutuality*					22	4.36(0.95)	19	3.37(0.83)	16	4.63(0.96)	19	4.05(1.31)

*The number of participant data available for each measure is indicated.*

*^a^One data-point each in NF1 and control group removed as >4 SD above mean.*

*^b^One data-point in control group removed as >4 SD above mean.*

### Trajectories of Cognitive, Motor and Behavioural Development

**FIGURE 2:**
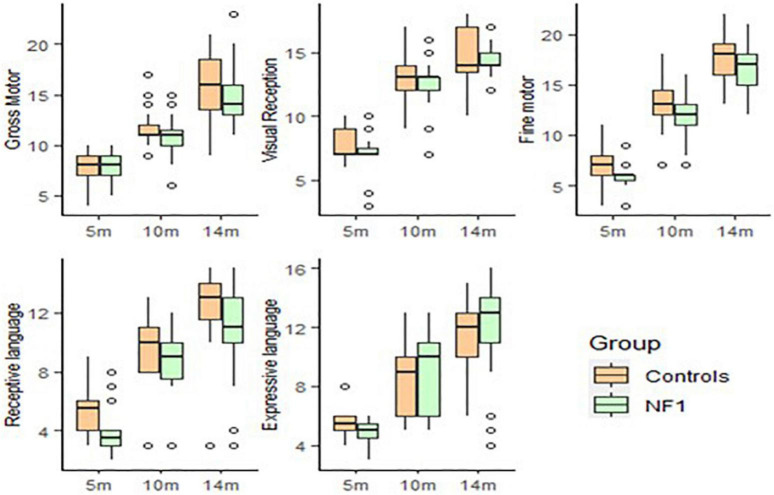
Box-whisker plots of the NF1 and control groups on the Mullen Scale of Early Learning.

Cognitive skills (Mullen) improved significantly with age in both groups [*t*(72) = 16.31, *p* < 0.001]. There were no intercept differences observed between the NF1 and control groups in Gross Motor [*t*(53) = 0.34, *p* = 0.73], Visual Reception [*t*(53) = −0.60, *p* = 0.55], Fine Motor [*t*(53) = −1.02, *p* = 0.31], Receptive Language [*t*(52) = −0.52, *p* = 0.60] or Expressive Language [*t*(53) = −0.75, *p* = 0.46]. No group differences were observed in developmental trajectories of Gross Motor [*t*(72) = −1.06, *p* = 0.29], Visual Reception [*t*(72) = 0.64, *p* = 0.52], Fine Motor [*t*(72) = 0.30, *p* = 0.77], Receptive[*t*(72) = −0.45, *p* = 0.66] or Expressive Language [*t*(72) = 0.97, *p* = 0.34]. Higher maternal education was associated with higher scores only on Visual Reception [*t*(72) = 2.56, *p* = 0.01] and Fine Motor [*t*(72) = 2.69, *p* = 0.01] skills (Figure 2).

For adaptive functioning (Vineland), there were no significant group differences at intercept in Communication [*t*(51) = 1.58, *p* = 0.11], Daily Living skills [*t*(51) = 0.18, *p* = 0.85], Socialisation [*t*(50) = −0.76, *p* = 0.45], Motor skills [*t*(51) = −0.20, *p* = 0.84] and overall Adaptive function [*t*(50) = 0.83, *p* = 0.41]. No significant group differences emerged with age in Communication [*t*(58) = −1.51, *p* = 0.14], Daily Living skills [*t*(57) = −0.27, *p* = 0.79], Socialisation [*t*(57) = −0.16, *p* = 0.87], Motor skills [*t*(57) = −0.79, *p* = 0.43] or overall Adaptive function [*t*(51) = −0.86, *p* = 0.39]. Higher maternal education was related to better Communication [*t*(58) = 2.53, *p* = 0.01], Socialisation skills [*t*(57) = 1.99, *p* = 0.05], Motor skills [*t*(57) = 2.02, *p* = 0.05] and Adaptive function [*t*(51) = 2.53, *p* = 0.01] but not with Daily Living skills [*t*(57) = 0.68, *p* = 0.50].

**FIGURE 3:**
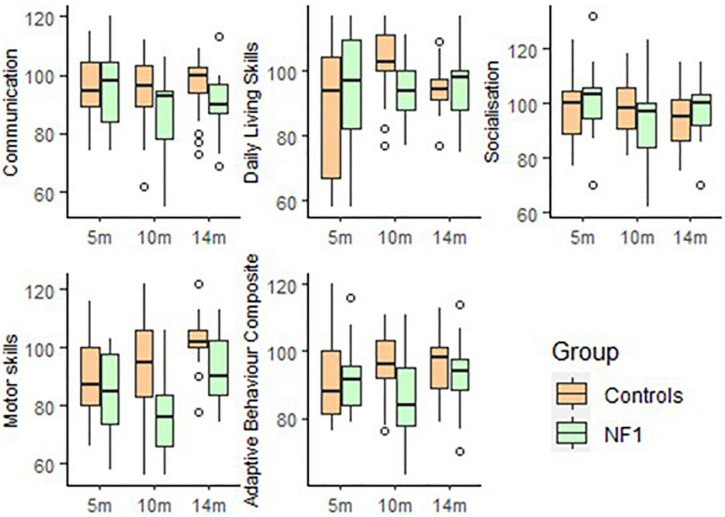
Box-whisker plots of the NF1 and control groups on the Vineland Adaptive Behaviour Scale.

**FIGURE 4:**
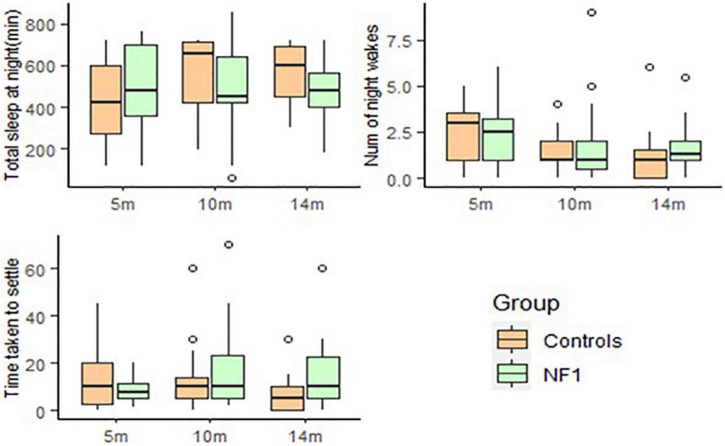
Box-whisker plots for the NF1 and control group on the Sleep and Settle Questionnaire.

Significant increase in Receptive [*t*(28) = 5.30, *p* < 0.001] and Expressive vocabulary [*t*(29) = 4.35, *p* < 0.001] as measured with the MCDI was observed with age. No group differences were observed at intercept in Receptive vocabulary [*t*(47) = 1.54, *p* = 0.13] or Expressive vocabulary [*t*(47) = 1.04, *p* = 0.30]. Similarly no significant group differences were observed in developmental trajectories of Receptive vocabulary [*t*(28) = −1.65, *p* = 0.11] or Expressive vocabulary [*t*(29) = −1.05, *p* = 0.30]. There was no effect of maternal education (Figures 3, 4).

For temperament (IBQ), there was a significant increase in Surgency [*t*(53) = 6.52, *p* < 0.001], decrease in Regulation Capacity[*t*(56) = −2.19, *p* = 0.03] with age but no significant change in Negative Affect [*t*(57) = 1.56, *p* = 0.12]. No significant group intercept differences were observed for Surgency [*t*(48) = 1.12, *p* = 0.27], Negative Affect [*t*(50) = −0.63, *p* = 0.53], or Regulation Capacity [*t*(50) = 0.80, *p* = 0.43]. Similarly group trajectories showed no significant differences in Surgency [*t*(53) = −1.40, *p* = 0.17], Negative Affect [*t*(57) = 0.49, *p* = 0.62], Regulation Capacity [*t*(56) = −0.05, *p* = 0.96]. Higher maternal education was associated with better Regulation Capacity [*t*(56) = 2.46, *p* = 0.02] but not with Surgency [*t*(53) = −0.28, *p* = 0.78] or Negative Affect [*t*(57) = 0.49, *p* = 0.08]. No significant group differences were observed on the AOSI total scores at 14 months [*t*(37) = −0.09, *p* = 0.93]. Higher maternal education had a marginally significant association with lower AOSI scores [*t*(37) = −1.97, *p* = 0.06].

The total amount of sleep at night [*t*(57) = 2.97, *p* = 0.004] increased with age, whilst the number of night awakenings [*t*(59) = −3.45, *p* = 0.001] and time taken to settle [*t*(56) = −2.35, *p* = 0.02] reduced with age. Total amount of sleep at night was marginally higher for NF1 group at intercept [*t*(49) = 1.94, *p* = 0.06] but significant group differences emerged over development with the NF1 group sleeping less than the control group [*t*(57) = −2.09, *p* = 0.04]. There were no group differences in the number of night awakenings at intercept [*t*(50) = −0.59, *p* = 0.55] or over development [*t*(59) = 0.55, *p* = 0.58]. No group differences were observed in the time taken to settle at intercept [*t*(49) = −1.49, *p* = 0.14] but the NF1 group took more time to settle over development [*t*(56) = 2.17, *p* = 0.03]. Infants with higher maternal education slept more at night [*t*(57) = 2.40, *p* = 0.02] but there was no effect of maternal education on the number of night awakenings [*t*(59) = −1.61, *p* = 0.11] or the time taken to settle [*t*(56) = −0.54, *p* = 0.59].

**FIGURE 5:**
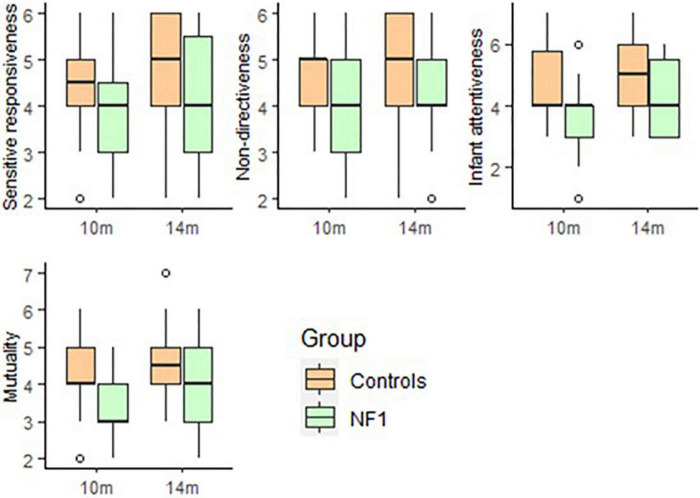
Box-whisker plots for the NF1 and control group on the Manchester Assessment of Caregiver–Infant Interaction (MACI).

For parent–child interaction (MACI) there were no significant group differences at intercept in parent Sensitive Responsiveness [*t*(46) = −1.42, *p* = 0.16] or Non-Directiveness [*t*(46) = −0.61, *p* = 0.55] but a trend of lower Infant Attentiveness [*t*(45) = −1.76, *p* = 0.08] and Mutuality [*t*(45) = −1.82, *p* = 0.07] was seen in the NF1 group. No significant group differences were observed over development in Sensitive Responsiveness [*t*(23) = 1.16, *p* = 0.26], Non-Directiveness [*t*(23) = 0.33, *p* = 0.74], Infant Attentiveness [*t*(23) = 1.34, *p* = 0.19] or Mutuality [*t*(23) = 1.27, *p* = 0.21]. There was no significant effect of maternal education on any of the MACI subscales (Figure 5).

The linear mixed modelling parameters are summarised in the online Supplementary Materials.

## Discussion

In this prospective longitudinal study, we describe the natural history of cognitive and behavioural development during infancy in NF1 infants, as compared to infants with no family history of neurodevelopmental disorders. To our knowledge, this is the first study to report developmental trajectories and examine the quality of parent–infant interaction in infants with NF1. Our results demonstrate that overall cognitive, motor and behavioural developmental trajectories of the NF1 group in the infancy period are similar to controls. Over the course of development, sleep difficulties in the NF1 cohort, including taking longer to settle to sleep and overall reduced sleep were noted. This data adds to the current body of literature by extending it to the infancy period and including prospective longitudinal assessments of infants with NF1.

In a previous prospective longitudinal study of NF1 children aged 21–40 months, Lorenzo et al. (2015) found diverging trajectories of cognitive development over time with lower scores in the NF1 group as compared to controls (Lorenzo et al., 2015). Cross-sectional studies confirm significant cognitive differences between the NF1 and control groups in the pre-school years, suggesting children with NF1 are at a significant disadvantage when they start school (Klein-Tasman et al., 2014). Contrary to findings described by these earlier studies, our results indicate that cognitive and motor function in the NF1 group in the infancy period are similar to controls, with subtle early differences emerging only in sleep functioning. We observed a trend of lower motor function at 10 and 14 months and parent–infant interactive differences mainly at 10 months in the NF1 group. Whilst behavioural differences were not prominent in this infancy period, our previous work in this cohort of infants found atypical low-level auditory processing in the NF1 group as compared to controls (Begum-Ali et al., 2021). Using an auditory habituation paradigm, we found that although infants with NF1 were able to discriminate between different auditory stimuli, there were developmental differences in the pattern of neural responses to auditory stimuli in the NF1 group as compared to controls, suggestive of auditory processing delays. We could speculate therefore that the NF1 mutations may affect low-level cognitive processes with cascading and cumulative effects on development over time (Karmiloff-Smith, 2006). Whilst more direct measures of brain function such as EEG (Begum-Ali et al., 2021) show early differences, it is possible that behavioural difference only become evident in the second year of life (Lorenzo et al., 2015). Further follow-up of this cohort will be important to (i) identify when the developmental trajectories in the NF1 group start to diverge from control groups, and (ii) to identify whether any early predictors might identify children most at risk for later cognitive and behavioural difficulties.

Consistent with other pre-school studies we find no difference on measures of temperament, behaviour or sensory processing (Klein-Tasman et al., 2014; Lorenzo et al., 2015). Both the direct assessment using the AOSI and parent reported VABS socialisation suggest that the developmental trajectories of social communication skills in NF1 are comparable to the control group during infancy. This is an interesting finding given recent reports of social communication deficits and ASD in children with NF1. In the general population, early signs of altered social communication in infants with later autism emerge between 12 and 18 months and may include reduced gaze following, deficits in social referencing (Cornew et al., 2012), repetitive play (Loh et al., 2007), motor and attentional atypicalities (Elsabbagh et al., 2013). It is plausible that these behavioural signs emerge later on in the developmental period for some children with NF1 who may then subsequently meet criteria for ASD.

Sleep disturbances particularly with sleep initiation and sleep–wake transitions are well known in NF1 both in animal model (Bai et al., 2018) and human studies (Johnson et al., 2005). We found that the NF1 infants take longer to settle and show overall reduced sleep over development as compared to the control group. In a cross-sectional study of 129 children aged 2–19 years compared to unaffected siblings, Licis et al. (2013) found that the NF1 group were significantly likely to have reduced nightly sleep durations, longer sleep onset latency and greater number of night awakening (Licis et al., 2013). The results in our study suggest that the sleep disturbances are evident in the infancy period based on parental report. Sleep disturbances impact on cognitive function, daytime functioning at school and may impact on mood and anxiety (Lavigne et al., 1999). Executive function skills, often impaired in NF1, may be particularly vulnerable to the effects of sleep disturbance. Simple early intervention approaches such as parental support and advice about sleep hygiene measures may be helpful in ameliorating sleep difficulties, which in turn may have a positive impact on cognitive development.

A significant finding in this study, both for the NF1 and control groups, was the impact of maternal education on cognitive and behavioural development. In a longitudinal study of cognitive development in children aged 6–18 years with NF1, Hou et al. (2020) found that higher parental education related to higher IQ, Maths, reading and cognitive scores (Hou et al., 2020). Two recent studies have also examined the cognitive and academic differences in children with inherited versus *de novo* NF1 mutations. One study suggested that lower IQ in the inherited NF1 group was largely mediated by lower socio-economic status in families with NF1 (Biotteau et al., 2020). Conversely another study found that having a parent with NF1 was related to lower academic and cognitive skills despite adjusting for socio-economic status (Geoffray et al., 2021). The authors speculate that the psychological and physical morbidity caused by NF1 may impact parenting ability and the provision of cognitive stimulation conducive to the development of cognitive abilities in children. Further research investigating the impact of parental NF1 on children development will be important, and early interventions may be targeted towards at risk NF1 populations; such as those with socioeconomic adversity or family history of NF1.

Limitations of this study include the relatively small sample size and the incomplete data for some of the participants over the three assessment periods. Unless confirmed via cord blood testing, NF1 is usually diagnosed based on clinical features, which become more prominent as the infant develops (Elsabbagh et al., 2013) with only 30% meeting the NIH criteria by 12 months (Pinti et al., 2021). It is therefore difficult to recruit infants younger than 12 months, particularly with *de novo* mutations into the infancy longitudinal study as the clinical features may not be apparent. Our analytic approach using linear mixed modelling provides a way to handle missing data, explicitly modelling change over time, accounting for correlations of observations within subjects.

In conclusion, this is the first longitudinal study that reports cognitive behavioural development in the infancy period in NF1. Our results suggest overall similarities in trajectories of cognitive, behavioural, sleep development in the NF1 and control groups. Given that children with NF1 are at substantially increased risk of cognitive and academic impairments by the time they start school, our work suggests that early clinical surveillance may be helpful, especially for those with a family history of NF1 and intervention approaches to promote sleep hygiene may help promote overall development.

## Data Availability Statement

Data is available from through the BASIS network through a set of data sharing procedures that comply with the ethical permissions under which this highly sensitive data was collected. Available at: https://www.basisnetwork.org/.

## Ethics Statement

The studies involving human participants were reviewed and approved by Greater Manchester Central Research Ethics committee (16/NW/0324) and National Research Ethics Service London Central Ethical Committee (16/EE/0167 and 06/MRE02/73). Written informed consent to participate in this study was provided by the participants’ legal guardian/next of kin.

## Members of the Eden–Staars Team

The STAARS team includes: Leila Dafner, Teodora Gliga, Amy Goodwin, Rianne Haartsen, Rebecca Holman, Sarah Kalwarowsky, Luke Mason, Laura Pirazzoli, and Chloë Taylor.

The EDEN team includes: *Manchester NF1 service*: Grace Vassallo, Emma Burkitt-Wright, Judith Eelloo, D. Gareth Evans, Siobhan West, Eileen Hupton, Lauren Lewis, and Louise Robinson; *Yorkshire Regional NF1 service:* Angus Dobbie, Ruth Drimer, and Saghira Malik Sharif; *Alder Hey NF1 clinic*: Jamuna Acharya and Zahabiyah Bassi; *Edinburgh Genetic Service:* Wayne Lam; *Sheffield NF1 clinic:* Alyson Bradbury, Neil Harrower, and Oliver Quarrell; *Newcastle NF1 service:* Helen Bethell, Rachel Jones, Susan Musson, Catherine Prem, and Miranda Splitt; *Sunderland NF1 clinic:* Karen Horridge; *Warrington NF1 clinic*: Shaheena Anjum; *Wirral University Hospitals NF1 clinic:* Christine Steiger.

## Author Contributions

SG contributed to the study conception and design, identified and recruited the participants, led the analysis, and drafted the manuscript and revisions. MWW led the analyses of parent–child interaction data, contributed to interpretation of findings, contributed to drafting of the manuscript, and commented on the revisions. JB-A led the fieldwork assessments, led data entry and initial analysis, led the quality control of phenotypic data, and contributed to drafting and critical review of the manuscript. AK-T contributed to the fieldwork assessments, data entry and initial analysis, and contributed to drafting and critical review of the manuscript. JG conceptualised the study and design, led the recruitment, and critically reviewed and revised the manuscript. MJ conceptualised and designed the study, and critically reviewed the manuscript. EJ conceptualised the study, contributed to the design, supervised the field-work, contributed to drafting of the manuscript, and critically reviewed and revised the manuscript. All authors contributed to the article and approved the submitted version.

## Author Disclaimer

The views expressed are those of the authors and not the funders, NIHR or UK Department of Health.

## Conflict of Interest

The authors declare that the research was conducted in the absence of any commercial or financial relationships that could be construed as a potential conflict of interest.

## Publisher’s Note

All claims expressed in this article are solely those of the authors and do not necessarily represent those of their affiliated organizations, or those of the publisher, the editors and the reviewers. Any product that may be evaluated in this article, or claim that may be made by its manufacturer, is not guaranteed or endorsed by the publisher.

## Abbreviations

NF1: neurofibromatosis type 1
ASD: autism spectrum disorder
ADHD: attention deficit hyperactivity disorder
Mullen: Mullen Scales of Early Learning
Vineland: Vineland Adaptive Behaviour Scales
IBQ: Infant Behaviour Questionnaire
ITSP: Infant-Toddler Sensory Profile
AOSI: Autism Observational Scale for Infants
SSQ: Sleep and Settle Questionnaire
MACI: The Manchester Assessment of Caregiver–Infant Interaction
MCDI: MacArthur-Bates Communication Development Inventory.
