# Supplementary material for: Early Developmental Trajectories in Infants With Neurofibromatosis 1
Source: Front Psychol. 2022 Jul 22;13:795951. doi: 10.3389/fpsyg.2022.795951 (PMC9355323; doi:10.3389/fpsyg.2022.795951)
Supplement: Supplementary file 1 [file Data_Sheet_1.DOCX]

**Supplemental materials**

Manchester Assessment for Caregiver- Infant Interaction (MACI)

Caregiver-infant was video recorded in which the caregiver was instructed to sit with their infant on a floor mat and play with their infant as they would normally do at home using an optional small set of supplied toys. The first 6-min of video (once the dyad had settled into play) were later rated by an independent trained rater, blinded to family information and study aims, using The Manchester Assessment of Caregiver-Infant Interaction.^1^ The MACI-Infant is a validated rating scheme that evaluates qualities of caregiver-infant interaction suitable for infants of 3-15 months of age, and has been extensively used in infants at risk of other neurodevelopmental disorder; i.e. autism.^2,3^ The coding scheme comprises eight 7-point scales, from which we focused on four scales of areas of parent-infant interaction that were affected among infants at familial risk of autism: (1) caregiver sensitivity (the degree to which the infant’s behaviour and state were met by prompt, appropriate and attuned responses to meet the infant’s immediate and developmental needs); (2) caregiver nondirectiveness (the lack of demanding, intrusive, critical and/or other controlling/restrictive behaviours or comments directed at the infant); (3) infant attentiveness to caregiver (the amount and quality of interest in, and engagement with the caregiver); (4) dyad mutuality (the amount and degree of dyadic reciprocity, closeness and sharedness). To determine inter-rater reliability, a random selection of over half the sample at each time point (23/41 at 10m; 22/35 at 14m) was blind rated by a further independent coder. Intraclass correlations using a single measures, absolute agreement definition at 10/14 months was as follows: caregiver sensitivity: r = .86/.81; caregiver directiveness r = .83/.75; Infant attentiveness: r = .81/.78; dyadic mutuality .80/.82; all p<0.001).

**References**

1. Wan MW, Brooks A, Green J, Abel K, Elmadih A. Psychometrics and validation of a brief rating measure of parent-infant interaction: Manchester assessment of caregiver-infant interaction. Int J Behav Dev 2017;41:542-9.

2. Green J, Charman T, Pickles A, et al. Parent-mediated intervention versus no intervention for infants at high risk of autism: a parallel, single-blind, randomised trial. Lancet Psychiatry 2015;2:133-40.

3. Wan MW, Green J, Elsabbagh M, et al. Quality of interaction between at-risk infants and caregiver at 12-15 months is associated with 3-year autism outcome. J Child Psychol Psychiatry 2013;54:763-71.
