# Supplementary material for: Early Developmental Trajectories in Infants With Neurofibromatosis 1
Source: Front Psychol. 2022 Jul 22;13:795951. doi: 10.3389/fpsyg.2022.795951 (PMC9355323; doi:10.3389/fpsyg.2022.795951)
Supplement: Supplementary file 2 [file Table_1.docx]

|  | **Mullen Gross motor** | | |  | **Mullen Visual reception** | | | |  | **Mullen Fine Motor** | | |  | **Mullen receptive language** | | | |  | **Mullen Expressive Language** | | | | |  | **VABS Communication** | | | |
| --- | --- | --- | --- | --- | --- | --- | --- | --- | --- | --- | --- | --- | --- | --- | --- | --- | --- | --- | --- | --- | --- | --- | --- | --- | --- | --- | --- | --- |
|  | *β* | SEM | *t* |  | *β* | SEM | *t* | |  | *β* | SEM | *t* |  | *β* | SEM | *t* | |  | *β* | SEM | | *t* | |  | *β* | | SEM | *t* |
| Intercept | 1.57 | 1.51 | 1.04 |  | 1.81 | 1.05 | 1.73 | |  | -2.53 | 1.23 | -2.05^*^ |  | 0.55 | 1.41 | 0.39 | |  | -0.37 | 1.26 | | -0.29 | |  | 74.90 | | 8.20 | 9.13^***^ |
| Age | 0.03 | 0.00 | 16.31^***^ |  | 0.02 | 0.00 | 13.43^***^ | |  | 0.03 | 0.00 | 19.67^***^ |  | 0.02 | 0.00 | 11.77^***^ | |  | 0.02 | 0.00 | | 9.99^***^ | |  | 0.00 | | 0.01 | 0.35 |
| NF1 Group | 0.44 | 1.27 | 0.34 |  | -0.66 | 1.10 | -0.60 | |  | -1.23 | 1.21 | -1.02 |  | -0.73 | 1.40 | -0.52 | |  | -1.03 | 1.37 | | -0.75 | |  | 11.33 | | 7.13 | 1.59 |
| Maternal Education | 0.12 | 0.38 | 0.32 |  | 0.62 | 0.24 | 2.56^*^ | |  | 0.80 | 0.29 | 2.69^**^ |  | 0.13 | 0.34 | 0.38 | |  | 0.38 | 0.28 | | 1.35 | |  | 5.13 | | 2.03 | 2.53^*^ |
| Age X NF1 group | -0.01 | 0.00 | -1.06 |  | 0.01 | 0.00 | 0.64 | |  | 0.00 | 0.00 | 0.30 |  | -0.00 | 0.00 | -0.45 | |  | 0.00 | 0.00 | | 0.97 | |  | -0.03 | | 0.02 | -1.51 |
|  | **VABS Daily living Skills** | | |  | **VABS Socialization** | | | |  | **VABS Motor skills** | | |  | **VABS Adaptive Behaviour Composite** | | | |  | **IBQ-Surgency** | | | | |  | **Negative Affectivity** | | | |
|  | *β* | SEM | *t* |  | *β* | SEM | *t* | |  | *β* | SEM | *t* |  | *β* | SEM | | *t* |  | *β* | SEM | | *t* | |  | *β* | | SEM | *t* |
| Intercept | 76.96 | 8.56 | 8.99^***^ |  | 86.71 | 8.21 | 10.56^***^ | |  | 62.49 | 9.29 | 6.72^***^ |  | 71.00 | 7.72 | | 9.20^***^ |  | 3.57 | 0.47 | | 7.52^***^ | |  | 3.96 | | 0.60 | 6.55^***^ |
| Age | 0.04 | 0.02 | 2.36^*^ |  | -0.01 | 0.01 | -1.20 | |  | 0.05 | 0.01 | 3.82^***^ |  | 0.02 | 0.01 | | 1.83 |  | 0.00 | 0.00 | | 6.51^***^ | |  | 0.00 | | 0.00 | 1.57 |
| NF1 group | 1.64 | 8.89 | 0.18 |  | 5.05 | 6.66 | 0.76 | |  | -1.57 | 7.92 | -0.20 |  | 5.09 | 6.09 | | 0.84 |  | 0.45 | 0.40 | | 1.12 | |  | -0.29 | | 0.46 | -0.63 |
| Maternal Education | 1.35 | 1.98 | 0.68 |  | 4.22 | 2.11 | 1.99^^^ | |  | 4.66 | 2.31 | 2.02^*^ |  | 5.00 | 1.98 | | 2.53^*^ |  | -0.03 | 0.12 | | -0.28 | |  | -0.28 | | 0.15 | -1.79 |
| Age X NF1 group | -0.01 | 0.02 | -0.27 |  | -0.00 | 0.02 | -0.16 | |  | -0.02 | 0.02 | -0.79 |  | -0.01 | 0.02 | | -0.86 |  | -0.00 | 0.00 | | -1.41 | |  | 0.00 | | 0.00 | 0.49 |
|  | ***Regulation capacity*** | | |  | ***SSQ Total sleep at night*** | | | |  | ***SSQ number night wakes*** | | |  | ***SSQ time taken to settle*** | | | |  | ***AOSI Total Score*** | | | | |  | | ***MACI Sensitive Responsiveness*** | | |
|  | *β* | *SEM* | *T* |  | *β* | *SEM* | | *T* |  | *β* | *SEM* | *t* |  | *β* | *SEM* | | *t* |  | *β* | *SEM* | | | *t* |  | | *β* | *SEM* | *t* |
| Intercept | 4.24 | 0.46 | 9.27^***^ |  | 127.33 | 111.98 | | 1.14 |  | 4.55 | 1.03 | 4.43^***^ |  | 30.32 | 12.51 | | 2.42^*^ |  | 17.99 | 4.22 | | | 4.26^***^ |  | | 3.10 | 1.21 | 2.57^*^ |
| Age | -0.00 | 0.00 | -2.19^*^ |  | 0.52 | 0.18 | | 2.97^**^ |  | -0.00 | 0.00 | -3.45^**^ |  | -0.03 | 0.01 | | -2.35^*^ |  | NA |  | | |  |  | | 0.00 | 0.00 | 0.50 |
| NF1 Group | 0.29 | 0.36 | 0.80 |  | 219.54 | 113.17 | | 1.94 |  | -0.544 | 0.91 | -0.60 |  | -15.29 | 10.24 | | -1.49 |  | -0.17 | 1.92 | | | -0.09 |  | | -2.10 | 1.48 | -1.42 |
| Maternal Education | 0.29 | 0.12 | 2.46^*^ |  | 65.01 | 27.13 | | 2.40^*^ |  | -0.42 | 0.26 | -1.61 |  | -1.76 | 3.25 | | -0.54 |  | -2.28 | 1.16 | | | -1.97 |  | | 0.30 | 0.21 | 1.46 |
| Age X NF1 group | -0.00 | 0.00 | -0.05 |  | -0.63 | 0.30 | -2.10^*^ | |  | 0.00 | 0.00 | 0.55 |  | 0.05 | 0.02 | | 2.17^*^ |  | NA |  | | |  |  | | 0.00 | 0.00 | 1.16 |
|  | ***MACI Non-directiveness*** | | |  | ***MACI Infant attentiveness*** | | | |  | ***MACI Mutuality*** | | |  | ***MacArthur Receptive vocabulary*** | | | |  | ***MacArthur Expressive***  ***vocabulary*** | | | | |  | |  | | |
|  | *β* | *SEM* | *t* |  | *β* | *SEM* | | *T* |  | *β* | *SEM* | *t* |  | *β* | *SEM* | | *t* |  | *β* | | *SEM* | | *t* |  | |  |  |  |
| Intercept | 2.72 | 1.11 | 2.46^*^ |  | 3.36 | 1.27 | | 2.64^*^ |  | 3.02 | 1.04 | 2.91^**^ |  | -119.24 | 51.66 | | -1.72 |  | -31.23 | | 9.13 | | -3.41^***^ |  | |  |  |  |
| Age | 0.00 | 0.00 | 0.83 |  | 0.00 | 0.00 | | 0.89 |  | 0.00 | 0.00 | 1.34 |  | 0.47 | 0.08 | | 5.30^***^ |  | 0.08 | | 0.01 | | 4.35^***^ |  | |  |  |  |
| NF1 Group | -0.82 | 1.34 | -0.61 |  | -2.77 | 1.58 | | -1.75 |  | -2.22 | 1.22 | -1.83 |  | 81.85 | 53.07 | | 1.54 |  | 11.15 | | 10.6 | | 1.04 |  | |  |  |  |
| Maternal Education | 0.37 | 0.20 | 1.90 |  | 0.13 | 0.22 | | 0.60 |  | 0.12 | 0.20 | 0.62 |  | -4.61 | 10.20 | | -0.45 |  | 1.48 | | 1.44 | | 1.03 |  | |  |  |  |
| Age X NF1 group | 0.00 | 0.00 | 0.34 |  | 0.00 | 0.00 | 1.34 | |  | 0.00 | 0.00 | 1.28 |  | -0.21 | 0.13 | | -1.65 |  | -0.02 | | 0.02 | | -1.05 |  | |  |  |  |

*β*, regression coefficients in the multilevel growth models; SEM, standard error of the mean; ^*^*p*≤0.05; ^**^*p*<0.01; ^***^*p*<0.001. ^p=0.051

**Supplementary Table 1**: Linear mixed modelling parameters for all the outcome measures
